# RNA Modifications in Pathogenic Bacteria: Impact on Host Adaptation and Virulence

**DOI:** 10.3390/genes12081125

**Published:** 2021-07-24

**Authors:** Laura Antoine, Roberto Bahena-Ceron, Heemee Devi Bunwaree, Martin Gobry, Victor Loegler, Pascale Romby, Stefano Marzi

**Affiliations:** Université de Strasbourg, CNRS, Architecture et Réactivité de l’ARN, UPR 9002, F-67000 Strasbourg, France; l.antoine@ibmc-cnrs.unistra.fr (L.A.); bahenaceron@unistra.fr (R.B.-C.); heemee-devi.bunwaree@etu.unistra.fr (H.D.B.); martin.gobry@etu.unistra.fr (M.G.); victor.loegler@etu.unistra.fr (V.L.); p.romby@ibmc-cnrs.unistra.fr (P.R.)

**Keywords:** RNA modifications, pathogenic bacteria, host-adaptation, stress adaptation, ribosomal RNA, tRNA, small non-coding RNA, mRNA

## Abstract

RNA modifications are involved in numerous biological processes and are present in all RNA classes. These modifications can be constitutive or modulated in response to adaptive processes. RNA modifications play multiple functions since they can impact RNA base-pairings, recognition by proteins, decoding, as well as RNA structure and stability. However, their roles in stress, environmental adaptation and during infections caused by pathogenic bacteria have just started to be appreciated. With the development of modern technologies in mass spectrometry and deep sequencing, recent examples of modifications regulating host-pathogen interactions have been demonstrated. They show how RNA modifications can regulate immune responses, antibiotic resistance, expression of virulence genes, and bacterial persistence. Here, we illustrate some of these findings, and highlight the strategies used to characterize RNA modifications, and their potential for new therapeutic applications.

## 1. Introduction

Bacteria are remarkably versatile organisms, which can survive and grow in numerous environmental niches on the planet. They modulate the expression of their genes to respond and adapt their growth to environmental stress such as temperature and pH shifts, nutrient availability, antimicrobials or dangerous chemical reactive species. This is particularly the case for pathogenic bacteria, which must adapt their metabolism to the host environment and ensure their survival facing the human/animal immune systems and antibiotic therapies. Bacteria can sense the environment directly via the effect that physical and chemical stresses might have on different macromolecules, or via signaling systems, very often two-component systems (TCS), transducing the stress signal from a specific sensor histidine kinase to a corresponding response regulator [1]. Responses are aimed at eliminating the stressor or its effects, at repairing damages or at inducing the escape from the stress, but sometimes the external stimuli are signals for switching the bacterial programmed lifestyles. Changes in cell physiology and behaviors involve extensive regulation of gene expression at transcriptional, post-transcriptional, translational, and post-translational levels. In these processes, an interplay between various TCS, transcriptional protein regulators, and regulatory RNAs orchestrates complex regulatory networks in order to link metabolism adaptation and virulence [2]. Because RNAs occupy a central position in translation (tRNAs, rRNAs, mRNAs), they actively contribute to these regulatory networks.

In bacteria, the rate of translation is modulated by multiple signals present in mRNAs affecting translation initiation, elongation, termination, and ribosome recycling [3,4,5,6]. The mRNA translation initiation signals can be masked or liberated by the binding of proteins or small regulatory RNAs (sRNAs), or by structural rearrangements mediated by metabolite binding or by physical cues changes, which also directly affect transcription and RNA folding [7,8,9,10,11,12]. Besides, codon usage specific for each gene (codon bias) is used in relation to variation of the tRNA pool to fine tune gene expression [13]. Moreover, many studies have emphasized the physiological importance of ribosome heterogeneity to rapid modulation of selective gene expression in response to environmental conditions [14,15]. These mechanisms are influenced by base or ribose modifications present in RNAs [16] adding another sophisticated layer of regulation. Stresses can also alter RNA modification states of various RNA species (i.e., rRNA, tRNA, sRNA and mRNA) with effect on translation rates, on RNA regulatory properties, or on codon recoding.

The number of newly detected modifications increases regularly [17]. They can be very simple like methylation or deamination or be more complicated. Some of them require the consecutive action of several modification enzymes and cofactors [18,19,20]. These modifications modulate the chemical and physical properties of the nucleotides and in turn the RNA functions (for a review see [21]). They might change any of the nucleotide interacting edges, which can potentially affect Watson-Crick base-pairing and non-canonical interactions [22]. For example, the A-form RNA helix favored by C3′-endo sugar pucker, is stabilized by 2′-OH methylation of ribose [23,24], while dihydrouridine (D) significantly destabilizes the C3′-endo sugar conformation providing structure flexibility [25]. Interestingly, D is predominantly found in psychrophile bacteria and archaea [25]. Methylation on the bases can influence hydrogen-bonding and stacking interactions [26]. Pseudouridine (Ψ) can shift between *syn/anti* conformations with relatively greater ease [27]. The dynamic nature and the regulatory functions of some of these RNA modifications [28] have generated a new field referred to ‘epitranscriptomics’ [29]. The characterization of the bacterial RNA modifications relies on cutting edge methodologies involving combination of mass spectroscopy (LC/MSMS) and RNAseq based methodologies. Nevertheless, identification of the epitranscriptome of pathogenic bacteria and its modulation upon stress and during the infection process is only in its infancy.

In this review, we illustrate several examples of the involvement of RNA modifications on the expression of virulence genes and in stress responses in different pathogenic bacteria. Even though these studies provide mechanistic explanations for only few cases, they already reveal a multitude of strategies developed by pathogenic bacteria to survive, persist, and fight against host immune defenses based upon the modulation of RNA modifications.

## 2. Technological Advances: Detecting RNA Modifications

A prerequisite to analyze the functions of RNA modifications is obviously to be able to detect the modified nucleosides, to quantify them, to map their localization within specific RNA. A detailed description of the available methods is outside the scope of the review. We will summarize the most employed approaches with their limits and advantages (Table 1), and details will be found in more specific articles [30,31,32,33].

Mapping of post-transcriptional RNA modifications using mass spectrometry (MS) existed for 30 years [34]. The precise measurement of molecular masses (less than 1 Da) provided the possibility to characterize known modified residues in tRNAs using liquid chromatography coupled to mass spectrometry (LC/MSMS). Two complementary MS approaches are routinely used to get a complete repertoire of modified nucleosides obtained either from tRNA extracts in a single experiment [35,36,37], or from tRNA fragments produced from specific endoribonuclease digestion to determine both the nature of modifications and their location in the tRNA sequence [38,39]. The analysis of a pure RNA or a class of RNAs hydrolyzed into nucleosides allows precise characterization and quantification of modifications, while oligonucleotide analysis allows precise localization of modified sites, but no quantification can be achieved. Moreover, the method does not allow complete sequence coverage but the use of RNases with different sequence specificity can increase it. Capillary electrophoresis coupled to MS (CE/MSMS) allows precise analysis of a wide range of molecules (reviewed in [40]) and can also be used for the characterization of tRNA modifications. Because capillary electrophoresis allows analysis of small oligonucleotide fragments, the combination of LC/MSMS and CE/MSMS leads to a better sequence coverage on pure tRNAs [41]. Nevertheless, one of the major limitations of these MS methods is the detection of Ψ, which is an isomer of uridine and for this reason is a mass-silent modification. However, MS analysis of Ψ can be done because they can be selectively modified by 1-cyclohexyl-(2-morpholinoethyl)carbodiimide metho-*p*-toluene sulfonate (CMCT) [42]. The carbodiimide (CMC) moiety of CMCT modifies the N1 of guanosine, the N3 of uridine, and the N1 and N3 of Ψ, but alkaline treatment at pH 10.3 easily removes it from them, with the exception of the N3 of Ψ, therefore producing a detectable mass increment of 252 Da [43]. More recently, Ψs have been also detected directly in RNA fragments by pseudo-MS^3^ (MS/MS/MS) analysis [44] thanks to the specific signature ion at *m/z* 207.041 for double dehydrated Ψ [45].

Various high throughput sequencing methods are often used to characterize specific modifications by reverse transcriptase (RT) signatures after chemical treatment. These methods do not need to purify the RNA prior to the analysis but they need bioinformatics data treatment and statistical evaluation of errors. NGS-based methods are often not quantitative, have high false-positive rates [46], and require multiple ligation steps and extensive polymerase chain reaction amplification during the library preparation, introducing undesired biases in the sequencing data [47]. Currently, customized protocols must be optimized for each RNA modification type leading to experimental design in which the RNA modification type to be studied is chosen beforehand. This limits the ability to characterize the plasticity of the epitranscriptome in a systematic and unbiased manner. A recent review described in details the RNAseq based approaches [31]. Noteworthy, the very recent development of single molecule direct RNA sequencing method by Oxford Nanopore Technologies (ONT, Oxford, UK), is promising for the analysis of modification landscape on a specific RNA sequence, including Ψs [48,49,50,51].

Combination of these highly complementary methods allows a precise localization and characterization of post-transcriptional modifications and their dynamics, a prerequisite to a better understanding of the roles of RNA modifications in bacterial adaptation and virulence.

## 3. Impact of RNA Modifications on Pathogenic Bacterial Stress Responses and Host Adaptation

### 3.1. rRNA Modifications

**Figure 1:**
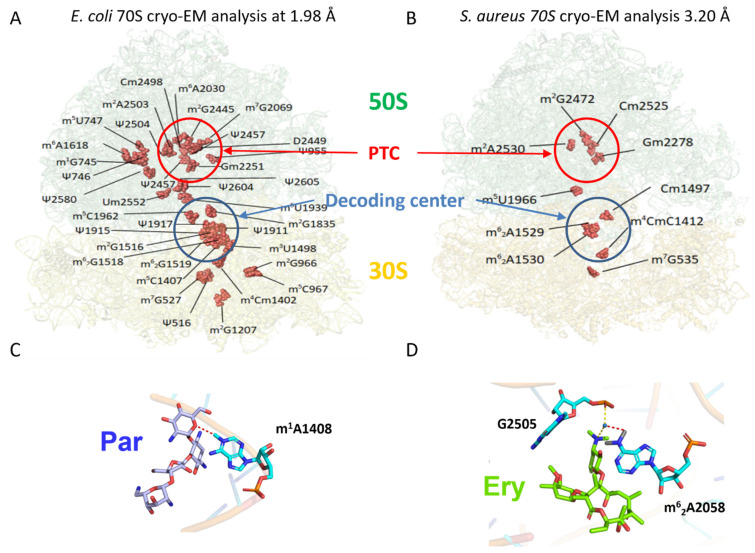
Natural ribosomal RNA modifications and additional modifications implicated in antibiotic resistance mechanisms as visualized by structural analyses. (**A**) Cryo-EM structure at 1.98 Å of *E. coli* 70S ribosome (pdb file 7K00). 11 and 24 RNA modifications (red spheres) could be visualized in the 30S (16S rRNA) and 50S (23S rRNA) subunits, respectively [88]. (**B**) Cryo-EM structure at 3.20 Å of *S. aureus* 70S ribosome (pdb file 6YEF). The limited resolution allowed the visualization of 4 modifications in the 16S rRNA and 6 in 23S rRNA [89]. PTC, Peptidyl Transferase Center on the large 50S subunit. (**C**) Mechanism of aminoglycoside (Par, paromomycin) resistance induced by methylation of A1408 (pdb file 5ZEJ [90]). The presence of the methyl group directly perturbs antibiotic interaction. (**D**) Mechanism of macrolide (Ery, erythromycin) resistance induced by dimethylation of A2058 (pdb file 6XHV [91]). The two methyl groups on A2058 prevent the coordination of a water molecule with G2505, which stabilizes erythromycin binding.

The ribosome is a powerful molecular machine and a huge ribonucleoprotein complex (2.3 MDa in bacteria), which performs the crucial task of translating the genetic information into proteins. The ribosome is also a ribozyme with its catalytic centers made by RNAs [80]. To carry out translation, the ribosome needs to balance between speed and accuracy and rRNA base modifications participate to fine tune ribosome structure and function. Indeed, in vitro reconstituted *Escherichia coli* ribosomes lacking rRNA modifications were severely defective in catalytic activity [81] and the ribosome assembly was also altered [82]. Numerous studies showed that the loss of rRNA modifications perturbs the active site structures [83,84], and causes altered rates and accuracy of translation [85]. In bacterial ribosomes, there are three major types of rRNA modifications: Ψ, methylation of the 2’-hydroxyl group of riboses (Nm), and methylation of base (mN) [86]. Even if specific role for several of these modifications was not yet attributed, they confer specific properties to the nucleotides. For instance, they can induce enhanced (Ψ) or decreased (D) base stacking, structure rigidity (Ψ and Nm) or flexibility (mN) to both single- and double-stranded regions with possible altered hydrogen-bonding [27,87]. These modifications are clustered in highly conserved areas devoted to decoding, peptidyl transfer, binding sites of A- and P- tRNAs, the peptide exit tunnel, and inter-subunit bridges [88,89], (Figure 1A,B).

Bacterial rRNA modifications have been investigated by mass spectrometry (MS), reverse transcriptase extension (RT), RNAseq, and structure analysis. Several high-resolution structures of 70S ribosomes have been achieved both by cryo-EM and X-ray studies. However, cryo-EM structures can be non-uniform in local resolution and confident assignment of modifications is often possible on their most structurally stable core. Assignment of 35 modifications on bacterial rRNAs was first obtained on the cryo-EM structure of *E. coli* 70S-EF-Tu-tRNA complex solved at 2.65–2.9 Å resolution [92]. This study provides clues on their roles in fine-tuning ribosome structure and function and in modulating the action of antibiotics. In this structure, the methyl group of nucleosides could be clearly visualized as extra densities, as well as the non-planar D at position 2449 of 23S rRNA, while Ψs were identified indirectly by polar residues within hydrogen-bonding distance of the N1 position. Besides, the rRNA modifications were unambiguously identified in the *Thermus thermophilus* and *E. coli* crystal structures at 2.3–2.5 Å resolution, respectively [93,94]. More recently, sub-stoichiometric modified nucleotides, like m^7^G527 and m^6^A1519 of the 16S rRNA, could be assigned on the structure of *E. coli* 70S solved at 2 Å resolution, and some antibiotic resistance mechanisms have been proposed [88] (Figure 1A). In the past three years, cryo-EM analyses of 70S [89] and 50S [95] from *Staphylococcus aureus* allowed the placing of 10 rRNA modifications (Figure 1B).

Because the literature about the functions of rRNA modifications is abundant, we have chosen specific examples in different bacteria, which illustrate the variety of their functional impact. For clarity, we refer to the *E. coli* numbering of the 16S and 23S rRNA sequences. In the decoding region of the small 30S subunit, several modified nucleotides contact tRNA or mRNA, or are close to positions known to be important for translation, contributing to the building of the decoding site. For example, the *E. coli* m^2^G966 contacts the P-site tRNA through stacking with nucleotide at position 34 of the tRNA [96]. Particularly, they stabilize the binding of initiator fMet-tRNA^fMet^ to the 30S pre-initiation complex prior to start codon recognition [97]. The correct folding of the 16S rRNA around the initiator tRNA helps to discriminate the initiator tRNA against other tRNAs. In this context, m^2^G966, m^6^_2_A1518 and m^6^_2_A1519 monitor the characteristic presence of the three consecutive GC pairs of the anticodon stem of the initiator tRNA [98]. In addition, m^4^Cm1402 and m^3^U1498 contact the P-site mRNA codon and play a role in fine-tuning the shape and function of the P-site [88] to increase initiation decoding fidelity [99]. In *S. aureus*, the m^4^Cm1402 modifications are important for infection since *rsmI* and *rsmH* genes encoding the two methylases of C1402 protect *S. aureus* from oxidative stress and restore translational fidelity [100].

On the large ribosomal subunit, *E. coli* Gm2251 and Um2552 are located in the P- and A- loops, respectively, to establish interactions with the CCA end of tRNAs in P- and A- sites. Gm2251 is conserved in all three kingdoms of life [101] whereas Um2552 is present in many bacteria except that in some Bacillus species, like *Bacillus subtilis* and *Bacillus stearothermophilus,* methylation at the neighboring G2553 is observed [102]. Absence of Um2552 modification (*rlmE*-deficient mutant) increases the flexibility of the nucleotides next to it, and induces 50S maturation delay, slowers subunit association and translocation rate [103]. Surprisingly, translation with unmethylated U2552 appears to be more accurate suggesting that a certain degree of recoding provided by methylation of this residue is important for cell physiology [104].

The ribosome and more generally the translation apparatus are main targets of antibacterial therapies [105,106,107,108]. However, bacteria are continuously evolving resistance mechanisms for antibiotics. Acquisition of additional rRNA modifications is one of the most direct marks of antibiotic resistance. For example, aminoglycosides target the 30S subunit to prevent translocation and A-site tRNA binding and promotes miscoding (Figure 1C), while macrolides bind to the nascent peptide exit tunnel on the 50S subunit to prevent peptide bond formation and translocation [109,110] (Figure 1D). Methylation of their rRNA target sites inhibits antibiotics binding [111]. Interestingly, the modification enzymes responsible for the resistance are often inducible and only synthetized when necessary for survival. For example, in *S. aureus* sub-inhibitory concentrations of the macrolide erythromycin (Figure 1D) stall ribosomes on the leader peptide for the methylase (*ermC*) responsible for the dimethylation of A2058 at the ribosomal peptide exit tunnel of the 50S subunit [112] (Figure 1D). This pausing induces the transcript to form a structure in which the Shine and Dalgarno sequence for *ermC* is exposed, allowing translation of *ermC* [112]. ErmC induced-dimethylation of A2058 prevents erythromycin binding (Figure 1D), but also results in inefficient translation of selected polypeptides [112]. It is interesting to note that S. aureus strains bearing m^6^_2_A2058 are not only erythromycin-resistant, but can also better escape host immune system, avoiding recognition by specific Toll-like receptors [113]. Other mechanisms of modifications inducing resistance but compromising bacterial fitness involve methylations of G1405 and A1408 in 16S rRNA, which are required for aminoglycoside resistance in Gram-negative bacteria (Figure 1C). In fact, these additional modifications interfere with the natural methylation at the neighboring C1407 residue (m^5^C1407) and decrease translation accuracy [114]. Conversely, antibiotic resistance could also arise from the lack of modifications at naturally occurring sites. Indeed, mutation of the *ksgA* gene encoding methyltransferase causes a defect of modifications at A1518 and A1519 in the 16S rRNA and induces kasugamycin resistance [115] accompanied by assembly defects and a cold sensitive phenotype [116]. Similarly, loss of methylation at m^7^G527, which is located near the mRNA decoding site has been shown to confer low-level streptomycin and neomycin resistance [117,118].

Finally, induction of rRNA modifications has been reported to be a key step in the ribosome reactivation during resuscitation of persistence state [119]. Indeed, antibiotic stresses together with a myriad of other stresses, sometimes induce the differentiation of a subpopulation of cells, which become dormant and multi-stress tolerant (persisters) [120]. This transient phenotype, which does not involve genetic changes, can be reverted via different mechanisms to re-activate ribosomes [121,122,123]. For instance, single-cell studies revealed that in *E. coli*, cells resuscitate and ribosome activity is resumed by the action of the RluD enzyme [119], which is responsible of 23S rRNA pseudouridine (5-ribosyl-uracil) modification at positions 1911, 1915, and 1917 [124].

### 3.2. tRNA Modifications

**Figure 2:**
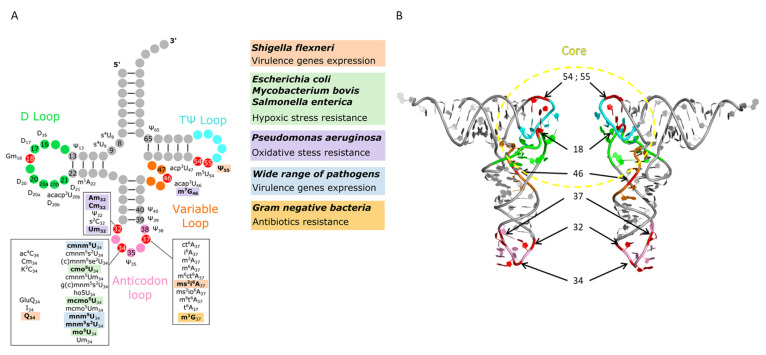
Pathogenic bacteria tRNA modifications involved in virulence and stress adaptation. (**A**) Secondary structure of tRNA. The nucleotides in the loops, where the majority of modifications accumulate, are colored as follow: D loop in green, anticodon loop in pink, variable loop in orange, and TΨ loop in light blue. The nucleotides for which the modifications are associated with a specific phenotype specified in the figure are in red. (**B**) Tertiary L-shape structure of tRNA. The 3D model corresponded to the crystal structure of *Saccharomyces cerevisiae* tRNA^Phe^_GAA_ (pdb file 1EHZ [133]). The same color code is used for the secondary (**A**) and tertiary (**B**) tRNA structures. The core domain of the tRNA comprises the D-, TΨ- and variable loops.

tRNAs are key molecules in translational process, their main purpose is to participate in protein synthesis by decoding the mRNA codons into the corresponding amino acids. They contain the largest number of modifications and the widest chemical diversity. Their modifications are known to improve tRNA decoding capacity, to decrease codon sensitivity, and to dictate codon choice and the maintenance of reading frame. Their localization concentrates in two hotspots—the anticodon loop and the tRNA core region, where the D- and TΨ -loops interact with each other (Figure 2) (reviewed in [125,126]).

Modifications in the tRNA core are important for the stability of tRNA structure and can contribute to temperature adaptation in thermophilic as well as in psychrophilic organisms [127]. Because they are involved in the tertiary interactions maintaining the L-shape, they are also expected to influence the binding of several proteins to the tRNA (i.e., EF-Tu, aminoacyl-tRNA synthetases, and anticodon modification enzymes) [128,129,130]. Different mutagenesis studies have demonstrated the role of these modifications in tRNA structure stabilization and their consequences on bacterial physiology during stress adaptation and on pathophysiology.

The 2′-O methylation of Gm18, present on several tRNAs and common to all Gram-negative bacteria, is lacking in most Gram-positive species despite the presence of putative *trmH*-like genes. Methylation of the ribose stabilizes G18 in its C3′-endo form increasing its rigidity [131,132] and promotes Gm18-Ψ55 base pairing (Figure 2).

The 5-methylation of U54 and the isomerization of U55 into Ψ55 enhance base stacking and stabilize the tRNA [134,135,136]. In the mesophilic *E. coli*, lack of the modification enzymes encoding *trmH* (Gm18), *trmA* (m^5^U54) and *truB* (Ψ55), reduces growth rate particularly at high temperature [137]. In the thermophilic *T. thermophilus*, adaptation to low temperature requires maintaining a sufficient flexibility of tRNAs. In this condition, methylation of the ribose of G18 is prevented but only if Ψ55 is present [138]. Moreover, the lack of some of these modifications induces changes in decoding and modulates frameshifting. In the pathogenic bacteria *Shigella flexneri*, Ψ55 is linked to the expression of several virulence factors, which are responsible of shigellosis, an intestinal infection causing diarrhea. Deletions of *trmH, trmA,* and *truB* genes in *S. flexneri* are associated with a reduction of the hemolytic activity and a decrease in the secretion system expression [137]. Furthermore, Gm18 is known to be responsible for TLR-7 dependent suppression of the immune response of dendritic cells, allowing a better tolerance of several enterobacteria by the host immune system [139,140,141].

The anticodon loop (ASL) is the other hotspot for modifications, which affect the geometry and physicochemical determinants governing the decoding process [142,143]. The anticodon regions of all tRNAs bind to their cognate mRNA codons on the ribosome with similar affinities, despite the fact that diverse codon-anticodon pairings should exhibit differences in base-pairing strengths [144]. The modifications in the tRNA anticodon loop compensate for potential binding differences and ensure uniform affinities of all tRNAs to their cognate codons [145]. Perturbations of these modifications selectively alter the spectrum of proteins during adaptation via rare codon usage and translational frameshifting [146]. Specifically, modifications of nucleotide at positions 37 and 34 (wobble) have a strong impact on maintaining the reading frame [146,147]. Modifications at positions 32 and 38, the first and last nucleotides of the loop, have also important consequences in modulating the affinity for specific codons, by inducing 32–38 base-pairing and reducing the size of the loop [148]. This interaction is coordinated with the identity of codons. Strong GC-rich codon-anticodon interactions are always balanced by a weaker 32–38 pairing and conversely, a weak AU-rich codon-anticodon interaction is coupled with a stronger 32–38 pairing [148]. Several studies have analyzed the link between modifications of anticodon loop residues and gene expression regulation. For example, the introduction of queuosine (Q) in the tRNA^Tyr^_GUA_, tRNA^Asn^_GUU_, tRNA^Asp^_GUC_ and tRNA^His^_GUG_ at the wobble position (G34) in Eukaryotes and Bacteria, permits efficient recognition of both NAC and NAU codons. This Q modification allows fine-tuning of translation and has been correlated with low-level translation of *Shigella* virulence factors, including the main transcriptional regulator VirF [149]. Its efficient translation depends on the presence of Q34 and 2-methylthio-N6-isopentenyladenosine (ms^2^i^6^A37) tRNA modifications. Deletion of either *tgt* (tRNA-guanine transglycosylase, for Q) or *miaA* (tRNA dimethylallyltransferase, for ms^2^i^6^A) genes leads to a less efficient synthesis of VirF in *S. flexneri* and reduces its pathogenicity [149]. In *E. coli*, it has been shown that ms^2^i^6^A is important for translation of RpoS, the general stress response alternative sigma factor, which is particularly rich in UUX-Leu codons over CUX-Leu codons [150]. Similarly, *S. flexneri virF* contains a high proportion of UUX-Leu codons [151]. Although the mechanism by which Q induces *virF* translation is not known, it is noticeable that putrescine or a combination of methionine and arginine metabolically related to putrescine, restore VirF expression of *S. flexneri tgt* mutant [152]. Since polyamine like putrescine can modulate translational fidelity and maintenance of reading frame [153,154], it is possible that translation misreading of *virF* could occur in absence of Q. Interestingly, in *E. coli* polyamine auxotroph mutant, translation rate drastically decreased and concomitantly Q level was reduced [155], suggesting that maintaining normal translation rate most probably requires Q modification at position 34 of some of the tRNAs.

Studies on U34 hypermodifications reveal the central role of wobble U modifications and associated enzymes in bacterial adaptation to environmental conditions and virulence in pathogens. Even if the precise mechanism remains unclear, in the absence of modifications, frameshift occurs resulting in the expression of alternate proteins [146,156]. Deletion of *gidA* and *mnmE*, encoding two enzymes of the 5-methylaminomethyl-2-thiouridine (mnm^5^s^2^U34) modification pathway, significantly reduced the colonization of *Salmonella Typhimurium* in liver and spleen accompanied by decreased invasion of epithelial cells and compromised ability to survive and replicate inside macrophages [157]. This effect can be explained in part by the fact that several colonization genes important for host cell invasion, including the T3SS genes *invAEG*, *spaPQ*, and *prgHJ*, were downregulated in the attenuated mutant strains [158]. In addition, the repression of several other proteins was observed in these mutant strains, such as the oxidoreductase Ygh, and the thiol peroxidase Tpx, which promote the survival of *S. Typhimurium* under the stressful conditions experienced within host macrophages [159]. The modified nucleotide mnm^5^s^2^U34 is also important for virulence in other bacteria. In *Streptococcus* species, Gram-positive bacteria responsible for a wide range of infections from skin infection to sepsis, GidA/MnmE modification enzymes are essential for acid stress and high temperature adaptation [160], for pathogenicity [161], reduced ability of adhesion and invasion in epithelial cells, and increased sensitivity to phagocytosis [162]. Transposon mutagenesis in *gidA* gene is leading to sensitivity to acidic conditions also in *Cronobacter sakazakii*, an opportunist pathogen causing neonatal meningitis, enterocolitis, septicemia, bloody diarrhea, and brain abscesses, decreasing its ability to growth in host digestive system [163]. One of the best-characterized example of host adaptation via the induction of specific tRNA modification at U34 to selectively translate codon-biased mRNAs for persistence genes, has been described for *Mycobacterium bovis* [164]. When mycobacteria species infect host lungs, they are phagocytized by alveolar macrophages, which are unable to kill and digest them. Consequently, the bacteria multiply and promote the formation of granulomas, which are symptomatic of chronic infections. Human granulomas lacking endothelial and blood vessel are highly hypoxic [165], and mycobacteria enter a quiescent state in which cell replication is halted or slowed [166]. In this condition, DosR, the master regulator of hypoxic bacteriostasis, mediates the expression of approximately 50 genes necessary for dormancy survival [167]. Translation activation of DosR requires that mo^5^U present at position 34 in tRNA^Thr^_UGU_ under aerobic conditions should be hypermodified leading to either cmo^5^U or mcmo^5^U. This hypermodification facilitates decoding of ACG codons, which are particularly abundant in *dosR* mRNA [164]. The rationale for this decoding has been described by structural analysis [168]. The presence of cmo^5^U34 induces a classical Watson-Crick base-pairing geometry involving the wobble position, which allows better stacking between U34 and purine 35, and as a consequence increases the stability of the codon-anticodon interaction.

In addition to wobble position, 2′-O-methylations of A, C, and U at position 32 by methyltransferases of the Trm family, have been shown to confer resistance to oxidative stress in *Pseudomonas aeruginosa*, allowing its survival during infection [169]. Hypomethylation of the 2′-O-ribose moiety at position 32, linked to reduced catalase activity, perturbs codon-anticodon interaction and results in translation insufficiency and misreading [170,171]. Thiolation of cytidine at position 32 catalyzed by TtcA, a [Fe-S] cluster enzyme, has been shown to play a role in the response to oxidative stress during infections caused by *P. aeruginosa* [172].

Finally, modifications of nucleotides in the variable loop contribute to host adaptation. For example, m^7^G46 catalyzed by tRNA guanine-N7-methyltransferase (*trmB*) is important for decoding efficiency of tRNA^Asp^_GUC_ and tRNA^Phe^_GAA_. In *P. aeruginosa*, loss of *trmB* has a strong negative effect on the translation of Phe- and Asp-enriched mRNAs, including those coding the major peroxide detoxifying enzymes, the catalases KatA and KatB, resulting in oxidative stress-sensitive phenotype [173]. Using tRNAseq and mass spectrometry performed on *Vibrio cholera* revealed specific modifications in various tRNAs that were not described in *E. coli* tRNAs [174]. These modifications include an acetylated acpU at either position 20 in the D-loop and at positions 46 or 47 in the variable loop. More interestingly, an editing process C-to- Ψ was for the first time identified at position 32 of the anticodon loop of tRNA^Tyr^. Although the physiological consequences of these specific features have to be defined, it is tempting to propose that RNA modifications contribute to the speciation of the bacteria and to the adaption of the organism to its specific niches.

### 3.3. sRNA Modifications

Small trans-acting regulatory RNAs (sRNAs) belong to a very heterogeneous class of RNAs regulating several processes including virulence gene expression, stress adaptation, and quorum sensing [175]. They exert their functions through specific interactions with diverse targets such as mRNAs, sRNAs, tRNA precursors, proteins or even with the ribosome [176]. The sRNAs are very different in length, sequence, structure and regulate gene expression using various mechanisms. So far only few examples of RNA modifications have been reported in bacterial sRNAs [177].

**Figure 3:**
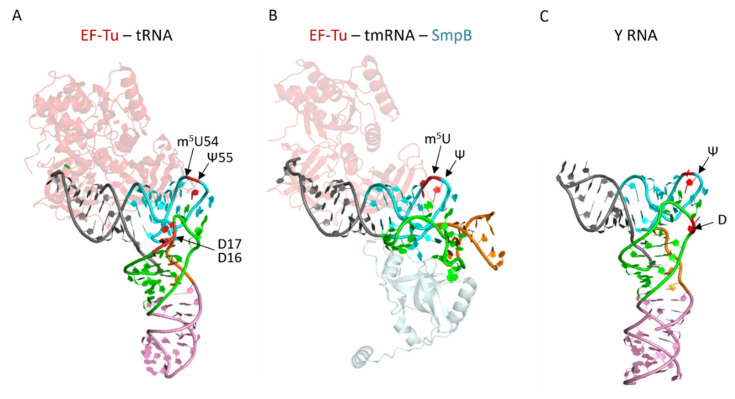
The structures of tmRNA and Y RNA mimic part of the tRNA structure. (**A**) The structure of EF-Tu-tRNA^Phe^ complex (with GDPNP, GTP analog) from *Thermus aquaticus* (pdb file 1TTT [189]). Each domain of the tRNA is colored: acceptor am in grey, TY-arm in light blue, the variable region in orange, the D-arm in green, and the anticodon-arm in pink. EF-Tu structure is represented in light pink. (**B**) The structure of the tmRNA fragment in complex with EF-Tu (with GDP and kirromycin antibiotic, in light pink) and SmpB (in light grey) from *Thermus thermophilus* (pdb file 4V8Q [190]). The regions of tmRNA mimicking tRNA are shown with the same color code as for the tRNA. (**C**) *Salmonella Typhimurium* Y RNA (YrlA, pdb file 6CU1 [191]). The regions of YrlA mimicking tRNA are shown with the same color code as for the tRNA.

The best examples of regulatory RNAs, where modifications have been identified, are tmRNA and Y RNA, both mimicking the tRNA structure (Figure 3). The tmRNA together with the small protein B (SmpB) is involved in trans-translation, the major and ubiquitous ribosome rescue system in bacteria [4]. This mechanism is taking place when ribosomes and tRNAs are stalled on problematic and often truncated mRNAs, which can lead to reduced translation [178]. Ribosome halting resulted from (i) chemical mRNA damages produced by environmental stresses, (ii) rare codons or problematic polypeptide stretches, (iii) drugs inducing translational misreading, non-programmed frameshifting, or stop codons readthrough, (iv) spurious RNase activity or cleavage of the mRNA in the A-site by RelE in response to starvation stress response, and from (v) abortive transcription termination [179,180,181,182,183]. In these situations, trans-translation operates to liberate the ribosome, and simultaneously to degrade the nascent truncated peptide [4]. A vacant ribosomal A-site is the signal recognized by the tmRNA/SmpB complex, which is delivered to the ribosome by the translation elongation factor EF-Tu. The tmRNA is characterized by two functional domains embedded into a conserved and complex structure, which are a tRNA-like domain (TLD) specifically aminoacylated with alanine and a mRNA-like domain (MLD) encoding a peptide tag targeting proteolysis [184,185]. TLD presents a typical tRNA TΨ-loop with two Ψs and one m^5^U [186]. These modifications most probably enhance tRNA structural mimicry and its use in translation as a canonical tRNA (Figure 3). Most probably that m^5^U54 in tmRNA is introduced by the SAM-dependent methyltransferase TrmA as it is for the tRNAs [187]. Although tmRNA has been shown to be essential for the expression of virulence factors during *S. Typhimurium* infection [188], to our knowledge no studies have been conducted to establish the role of its modifications in bacterial adaptation or infection.

The non-coding Y RNAs are present in both eukaryotes and in several bacteria including some pathogens [192]. Bacterial Y RNA, known as YrlA (Y RNA-like A) RNA, is a modular RNA of variable length (between 90–150 nucleotides) characterized by a large stem involving pairings between nucleotides at the 5′ and 3′ ends (Figure 3) and a tRNA-like domain [193]. This domain shows high similarities to the D, TΨ and acceptor arms of tRNA (Figure 3). Due to this structure similarity, YrlA is a substrate for two tRNA modification enzymes DusA and TruB, which introduce D and Ψ in the D and TΨ loops, respectively [194]. The basal stem of YrlA tethers the monomeric ring Rsr protein similar to eukaryotic Ro60 protein, while the effector tRNA-like domain binds the ring-shaped 3′ to 5′ exoribonuclease polynucleotide phosphorylase (PNPase), forming a double-ringed RNA degradation machine called RYPER [193]. Although the functions and mechanism of RYPER are still under investigation, Rsr and YrlA have been shown to alter PNPase substrate specificity to preferentially direct the degradation of structured RNAs (including rRNAs) [193,195]. By altering the levels of specific RNA populations, RYPER has been proposed to be involved in stress responses, such as UV irradiation or prolonged stationary phase [196,197]. In *Salmonella enterica*, expression of YrlA appears to be confined to certain infection stages [198]. Surprisingly, it was reported that lupus autoimmunity might be triggered and sustained by commensal bacteria expressing Rsr RNPs [199]. Noteworthy, in some bacteria including *S. Typhimurium*, *rsr* and Y RNA genes are located within an “RNA repair” operon including *rtcA*-*rtcB* encoding RNA cyclase and RNA ligase, respectively [193]. The transcription of the whole operon is activated by tRNA fragments resulting from the SOS response to DNA damage [200]. tRNA fragments also accumulate when tRNAs are hypomodified, such as in *ΔtruA* strain missing Ψ at positions 38, 39, and 40 in the anticodon arm of some tRNAs [201]. Since tRNA fragments are both natural substrates of PNPase and of RtcB religation, it is possible that assembly of RYPER protects them from degradation and that the expression of RtcB could restore tRNAs from halves and translation [200]. Interestingly, *E. coli* RtcB re-ligates a 16S rRNA 3′ fragment containing the anti-Shine-Dalgarno sequence cleaved by the MazF toxin [202]. This indicates a tight link between RNA modification levels, translation regulation, and RNA metabolism in response to stress.

Another example of sRNA modification in pathogenic bacteria derives from a transcriptome-wide profiling of m^6^A distribution in *P. aeruginosa*. Methylation sites were found present in two major sRNAs, RsmY and RsmZ [203], which sequester the regulatory protein RsmA to control its activity which is associated with acute and chronic virulence phenotypes [12,204]. The involvement of these modifications in the regulatory mechanisms of RsmY/Z has not been studied yet.

Recently, one of the best characterized sRNA in *S. aureus*, an opportunistic pathogen causing a large variety of infections, has been shown to contain at its 5′ end a peculiar modification which is co-transcriptionally introduced [205]. The expression of RNAIII is under the control of a two-component system, which senses bacterial cell density, to orchestrate the regulation of virulence gene expression [206,207]. This dual RNA codes for the cytotoxic δ-hemolysin peptide, while its 5′ and 3′ UTRs act as antisense RNA to regulate at the post-transcriptional level the expression of virulence genes associated with infectious diseases [207]. Hence, through basepairing interactions with its target mRNAs, it represses several cell wall associated proteins involved in adhesion and tissue colonization, and the transcriptional repressor of toxins Rot while it activates directly or indirectly the synthesis of many secreted proteins and toxins required for infection dissemination. A new RNAseq based approach has been developed to detect the incorporation of NAD, the nicotinamide-adenine dinucleotide, at the 5′ ends of RNA transcripts [208]. This study found only few *S. aureus* transcripts containing this 5′ cap, including RNAIII. In Gram-negative bacteria, NAD has been reported to have a stabilization effect protecting the RNA from the 5′ processing enzyme RppH, which produces monophosphate at the end of RNAs that are substrates of RNase E [208]. In *B. subtilis*, NAD protects RNAs from the exonucleolytic activity of RNase J1/J2 [209]. In *S. aureus*, the presence of NAD in RNAIII does not induce its stabilization nor affects its structure, but it has important consequences on pathophysiology. By a yet unknown mechanism, the NAD modified RNAIII leads to a decreased expression of δ-hemolysin and reduced cytotoxicity [205].

### 3.4. mRNA Modifications

Besides the classical 5′-cap m^7^G modification of eukaryotic mRNAs, other modifications have been described, including the 6-methyladenosine (m^6^A), the 5-methylcytosine (m^5^C), inosine (I) derived from adenine deamination and pseudouridine (Ψ). These modifications, which are present within 5′- and 3′-untranslated regions (UTRs) and the coding sequences of mRNAs, contribute to fine tune gene regulation [210,211,212,213,214,215,216]. In bacteria, the presence and the roles of mRNA modifications is relatively unexploited in pathogenic bacteria. In bacterial mRNAs, the presence of non-canonical 5′ ends has recently been reported for a subset of mRNAs. As described above, co-transcriptionally introduced 5′ NAD cap has also been detected in some *E. coli* mRNAs and is thought to modulate mRNA stability and translation efficiency [208,217]. Recently, NAD cap has been found in mRNAs expressed in *B. subtilis* spores, a dormant state developed in response to different stresses [218]. Its role in these mRNAs remains to be analyzed. Other studies have shown additional types of 5′ capping directly incorporated into mRNAs during transcription initiation, which might increase mRNA stability. For example, dinucleoside tetraphosphates, very often Np4A, have been reported in *E. coli* [219]. Interestingly, the incorporation of such dinucleoside depends on their cellular concentration, which increases in some stress conditions such as heat shock [220] and oxidative stress [221].

Bacterial mRNAs can also contain post-transcriptional modifications. In *E.* coli, Ψs have been detected in some mRNAs. They can be located at specific codons and as a consequence they alter translation speed or mRNA decoding [222], and at stop codons to induce nonsense suppression [223,224]. Ψs have been proposed to influence the kinetics of RNase E-directed degradation in Gram-negative bacteria [225]. Another abundant mRNA modification is m^6^A, which has been detected in genes involved in energy metabolism in Gram-negative bacteria, including pathogenic *P. aeruginosa* [203]. Very recently, it has been shown that m^6^A reduces both sense and stop codon reading accuracy [226,227]. The molecular explanation for this decoding perturbation could be due to the formation of less stable codon-anticodon interactions with cognate tRNAs [226]. Indeed, when compared with unmodified A, m^6^A forms less stable base-pairing with uridine (U) and destabilizes local RNA structures and short duplexes [228,229]. It remains to be studied whether these modifications in mRNAs vary significantly in response to metabolic changes or stresses.

Adenosine deamination is most probably the major modification in mRNAs, which can directly influence the activity of the synthesized protein. Adenosine-to-inosine (A-to-I) mRNA editing is catalyzed by the tRNA adenosine deaminase enzyme TadA, which recognizes stem-loop structures resembling the anticodon arm of tRNA^Arg^ [230]. Because inosine (I) is recognized as guanosine (G) by the translational machinery, A-to-I editing expands the decoding rules resulting in protein diversification. In *E. coli* and in the two pathogenic bacteria *Klebsiella pneumonia* and *Yersinia enterocolitica*, recoding tyrosine (UAC) to cysteine (UGC) has been reported in mRNAs encoding self-killing toxins like HokB [230]. The presence of this cysteine increases HokB toxicity and induces cellular growth arrest in response to starvation as a function of cellular density via membrane depolarization [230]. By doing so, it mediates antibiotic tolerance leading to persistence [231]. Since bacteria persistence is characterized by the existence of sub-populations of bacteria that are tolerant to an antibiotic treatment with a fitness cost [232], the heterogeneous RNA editing levels of *hokB* mRNA could promote non-genetic cell heterogeneity [233].

## 4. Conclusions and Perspectives

The review has presented several examples which unveil the diversity of functions of RNA modifications in translation and RNA degradation in bacterial pathogens. Because technologies are constantly improving, it is expected that the whole set of modifications will be mapped in RNA molecules as well as their quantifications. This will be the prerequisite to better study their dynamics upon stress or during bacterial infection. Further works correlating the modifications with the characterization of their protein co-factors (referred as writers, readers, and erasers) will be another step necessary to get an in-depth overview of their functions and the physiological consequences on bacterial pathogens. Many questions still remain to be addressed: what is the extent of chemical modifications diversity and complexity among evolutionary distant bacteria? What are the consequences of RNA modification defects on bacterial physiology and pathogenesis? Are there specific classes of bacterial sRNAs and mRNAs that could be modified extensively as it is for tRNAs and rRNAs? Many mRNAs carry regulatory tRNA-like elements such as found in the T-Box regulatory elements [129] or in the translational operator region of *thrS* mRNA [234]. As it was shown for tmRNA or Y-RNA, such tRNA-like motifs might be recognized by specific modification enzymes.

Some of the data suggested that some tRNA modifications are species-specific. Hence, it is expected that modifications might contain some metabolites issued from specific metabolic pathways resulting from the bacterial adaption to its ecological niches. Meanwhile during the infection process, the pathogens have to face metabolic burden and should counteract the host defenses mechanisms. It would not be so surprising that some of the modifications might be acquired or disrupted during the infection process with consequences on the bacterial proteome. The functional studies of the modification enzymes might also lead to biotechnological applications, to design gene reprogramming or new tools for RNA studies, or to select novel anti-microbial strategies. Many of these studies will require the development of more simple technologies, such as the direct RNA sequencing methodologies, to facilitate the mapping and the discovery of RNA modifications among bacterial species. Although many studies have been done on tRNA and rRNA modifications, we are still far to fully appreciate the impact of modifications on RNA functions. We are only at the beginning of the tip of the iceberg.

## Figures and Tables

**Table 1:** Summary of main techniques for the detection of RNA modifications.

Methods			Modifications Detected	Quantification	Genome Wide	Positional Information	Remarks (Pros/Cons)
Structure Determination	X-ray Cristallography		All modifications	**✕** **/** **✓**	**✕**	**✓**	Difficult to obtain crystals
	Cryo Electron Microscopy						Heterogeneous resolution
	Nuclear Magnetic Resonance						Size limit
LC/MSMS	Nucleoside analysis	DMRM [34]	Known modifications	**✓**	**✓**	**✕**	Fragmentation pattern and retention time of modifications must be known
		NLS [34]	Various modifications	**✓**	**✓**	**✕**	NLS is less suitable for quantification than DMRM
	Fragment analysis	With a reference (SILNAS/CARD/ SILCARD) [52,53]	Known modifications	**✓**	**✕**	**✓**	Relative quantification can be assessed with reference in vitro RNA
		Without reference (RNase digests) [39]	Known modifications	**✕**	**✕**	**✓**	Determination of base composition and localization by comparing mass-spectrometry results with expected RNase fragments
NGS-based methods	RNA deep-sequencing direct method		A-to-I [54]	**✓**	**✓**	**✓**	To be accompanied by DNA sequencing to distinguish editing events from SNPs
			Methylations [55]	**✕**	**✕**		Based on RT stops or misincorporations
	Nanopore RNA sequencing [56,57]		m^6^A, m^5^C, A-to-I, Ψ and others	**✓**	**✓**		Based on the use of systematic base-calling ‘errors’ caused by the presence of RNA modifications. Software is still in development
	Indirect methods: chemical treatments	ICE-Seq [58,59]	A-to-I	**✓**	**✓**		No need of DNA seq
		Bisulfite-Seq [60]	m^5^C				
		Riboxi-Seq [61]	Nm				
		RiboMethSeq [62,63]					
		Pseudo-Seq [64]	Ψ				
		Ψ-Seq					
		PSI-Seq [65]					
		HydraPsi-Seq [66]					
		SLAM-Seq [67]	s^4^U				
		ARM-Seq [68]	m^1^A, m^3^C, m^1^G				
		TRAC-Seq [69]	m^7^G				
		AlkAniline-Seq [70]	m^7^G, m^3^C, D				
	Indirect methods: IP	miCLIP [71]	Methylation	**✓**	**✓**		
		m^6^A-Seq [72]	m^6^A				
		meRIP-Seq [73]					
		m^6^A-LAIC-Seq [74]					
		Nm-Seq / 2OMe-Seq [75]	Nm				
		acRIP-Seq [76]	ac^4^C				
		NAD capture-Seq [77]	5’-NAD cap				
Affinity gel electrophoresis	Mercury-sulfur affinity [78]		s^2^U, s^4^U	**✕**	**✕**	**✕**	APM treatment (Acrylo-aminophenylmercuric chloride)
	Boronate affinity [79]		NAD- or FAD-modified RNAs	**✓**	**✓**	**✕**	APB treatment (Acryloylaminophenyl boronic acid); fast screening (easy and quick); quantification possible as per intensity of bands

**✕** = not available; **✓** = available.

## Data Availability

Not applicable.
